# Antibacterial and Antibiofilm Effects of Photodynamic Treatment with *Curcuma* L. and *Trans*-Cinnamaldehyde against *Listeria monocytogenes*

**DOI:** 10.3390/molecules29030685

**Published:** 2024-02-01

**Authors:** Aleksandra Zimińska, Izabela Lipska, Joanna Gajewska, Anna Draszanowska, Manuel Simões, Magdalena A. Olszewska

**Affiliations:** 1 Department of Food Microbiology, Meat Technology and Chemistry, The Faculty of Food Science, University of Warmia and Mazury in Olsztyn, Plac Cieszyński 1, 10-726 Olsztyn, Polandjoanna.gajewska@uwm.edu.pl (J.G.); 2 Department of Human Nutrition, The Faculty of Food Science, University of Warmia and Mazury in Olsztyn, Słoneczna 45F, 10-718 Olsztyn, Poland; anna.draszanowska@uwm.edu.pl; 3 LEPABE—Department of Chemical Engineering, Faculty of Engineering, University of Porto, 4200-465 Porto, Portugal; mvs@fe.up.pt; 4 ALiCE—Associate Laboratory in Chemical Engineering, Faculty of Engineering, University of Porto, Rua Dr. Roberto Frias, 4200-465 Porto, Portugal

**Keywords:** *Curcuma* L., *trans*-cinnamaldehyde, photodynamic inactivation, biofilms, *Listeria monocytogenes*, flow cytometry

## Abstract

Photodynamic inactivation (PDI) is a highly effective treatment that can eliminate harmful microorganisms in a variety of settings. This study explored the efficacy of a curcumin-rich extract, *Curcuma* L., (Cur)- and essential oil component, *trans*-cinnamaldehyde, (Ca)-mediated PDI against *Listeria monocytogenes* ATCC 15313 (Lm) including planktonic cells and established biofilms on silicone rubber (Si), polytetrafluoroethylene (PTFE), stainless steel 316 (SS), and polyethylene terephthalate (PET). Applying Ca- and Cur-mediated PDI resulted in planktonic cell reductions of 2.7 and 6.4 log CFU/cm^2^, respectively. Flow cytometric measurements (FCMs) coupled with CFDA/PI and TOTO^®^-1 staining evidenced that Ca- doubled and Cur-mediated PDI quadrupled the cell damage. Moreover, the enzymatic activity of Lm cells was considerably reduced by Cur-mediated PDI, indicating its superior efficacy. Photosensitization also affected Lm biofilms, but their reduction did not exceed 3.7 log CFU/cm^2^. Cur-mediated PDI effectively impaired cells on PET and PTFE, while Ca-mediated PDI caused no (TOTO^®^-1) or only slight (PI) cell damage, sparing the activity of cells. In turn, applying Ca-mediate PDI to Si largely diminished the enzymatic activity in Lm. SS contained 20% dead cells, suggesting that SS itself impacts Lm viability. In addition, the efficacy of Ca-mediated PDI was enhanced on the SS, leading to increased damage to the cells. The weakened viability of Lm on Si and SS could be linked to unfavorable interactions with the surfaces, resulting in a better effect of Ca against Lm. In conclusion, Cur demonstrated excellent photosensitizing properties against Lm in both planktonic and biofilm states. The efficacy of Ca was lower than that of Cur. However, Ca bears potent antibiofilm effects, which vary depending on the surface on which Lm resides. Therefore, this study may help identify more effective plant-based compounds to combat *L. monocytogenes* in an environmentally sustainable manner.

## 1. Introduction

*Listeria monocytogenes* (Lm) is known to contaminate food during production processes, leading to foodborne illnesses. The food-processing environment (FPE) may be a source of contamination due to the ability of Lm to persist in FPE for an extended time [1]. This persistence is attributed to the difficulty in removing cells from hard-to-clean sites and/or biofilm formation, where the cells are more resistant to cleaning and disinfection and other treatment conditions [2]. Therefore, it is crucial to understand the response of Lm to specific treatments to develop better mitigation strategies.

Photodynamic inactivation (PDI) is a rising antimicrobial strategy that relies on a photochemical reaction that requires light and photosensitizers (PSs) in the presence of oxygen. The treatment works by activating PSs with specific wavelengths of light, which generate reactive oxygen species (ROS) with strong oxidation [3]. Unlike other treatments, PDI does not produce toxic chemicals, and the only energy required is the light source. Moreover, the chance of building up microbial resistance is low because of its multi-target nature [4]. PDI has been successfully used in medicine, dentistry, and the environment [5]. In food science, the use of PDI is gaining importance as a potential solution for eliminating foodborne pathogens. There is an interest in investigating the effects of PDI on these pathogens and the factors that can influence its effectiveness. It is crucial to evaluate the efficacy of PDI under different photosensitizing conditions. However, a comprehensive comparison of the susceptibility of various pathogens to PDI using different photosensitizers is yet to be completed.

Some PSs from natural sources have been suggested. *Curcuma* L. is an example of a source with various bioactive properties, such as anti-inflammatory, antioxidant, antimicrobial, and anticancer actions [6]. The raw material contains about 2% curcuminoids, including curcumin (diferuloylmethane), bisdemethoxycurcumin, and demethoxycurcumin. Nevertheless, curcumin is *Curcuma*’s vital fraction, responsible for all its biological activities [7]. Additionally, curcumin acts as a photosensitizer, absorbing photons and inducing reactions that can lead to the formation of ROS [6]. These ROS can cause cell toxicity by interacting with cell membranes or proteins [8]. Under light irradiation at 400–500 nm, the photosensitizing potential of curcumin can be effectively determined. Lately, there has been a growing interest in researching the potential applications of curcumin and PDI in enhancing food safety [9,10,11,12].

*Trans*-cinnamaldehyde is a phenylpropanoid that is present in edible essential oils like cinnamon bark. In PDI, *trans*-cinnamaldehyde can act as a pro-photosensitizer, magnifying ROS production, referred to as an oxidative stress-amplified agent [13]. Its photosensitizing properties to combat foodborne pathogens have not been thoroughly studied yet.

This study evaluated the antibacterial and antibiofilm effects of *Curcuma* L. and *trans*-cinnamaldehyde via the photodynamic action induced by blue light in *Listeria monocytogenes*. The bactericidal efficiency of edible photosensitizers was tested against planktonic cells and biofilms established on various surfaces, such as silicone rubber (Si), polytetrafluoroethylene (PTFE), stainless steel 316 (SS), and polyethylene terephthalate (PET). We investigated the treatment mechanism by analyzing enzymatic activity and membrane damage to Lm through flow cytometry using CFDA/PI staining. To better understand the cell membrane damage status, we also employed TOTO^®^-1 staining. We then used PCA analysis to resolve how the culture and treatment conditions impact Lm viability. The interactions between Lm and surfaces and the hydrophobicity of Lm on surfaces were analyzed to see if the surface on which Lm resides affects its behavior.

## 2. Results

### 2.1. Antibacterial and Antibiofilm Activity

**Figure 1:**
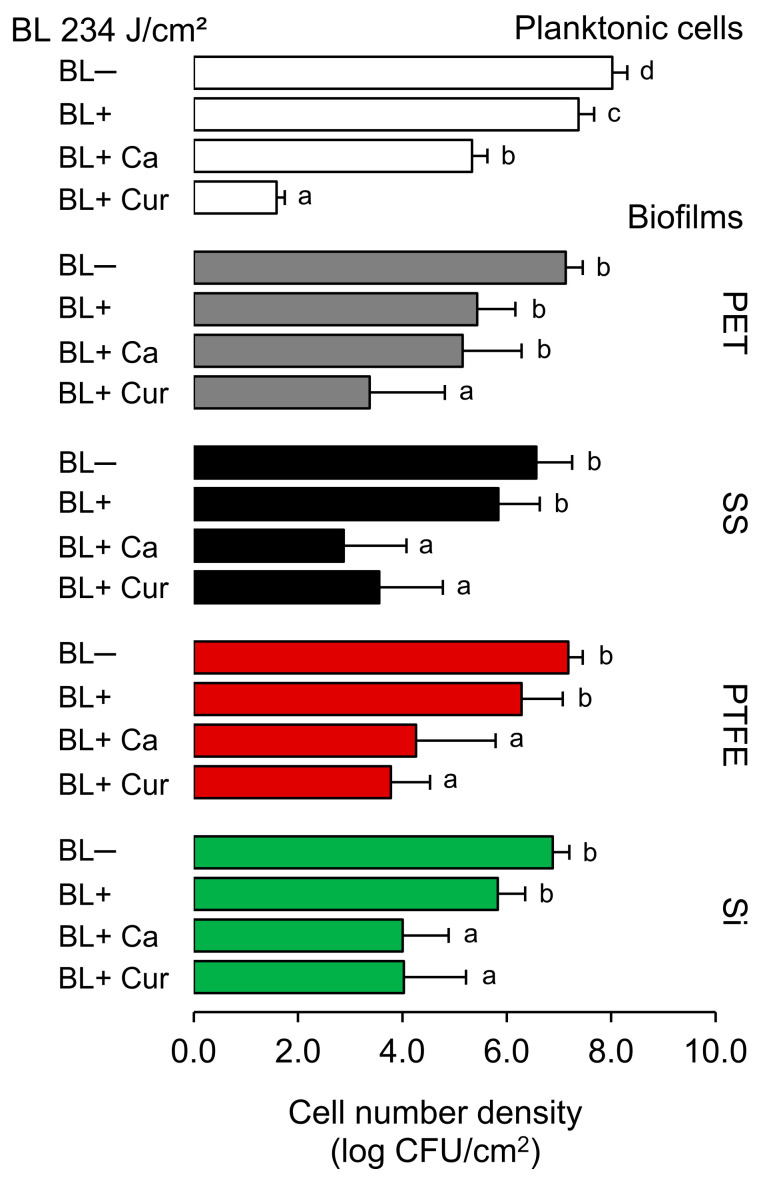
Effect of photodynamic treatments at 234 J/cm^2^ against *Listeria monocytogenes* ATCC 15313 planktonic cells and established biofilms on different surfaces, including silicone rubber (Si), polytetrafluoroethylene (PTFE), stainless steel 316 (SS), and polyethylene terephthalate (PET). Different letters indicate a significant difference within each culture variant (*p* < 0.05). BL− and BL+ represent the absence and the presence of blue light illumination. Ca and Cur stand for *t*-cinnamaldehyde and *Curcuma* L., respectively.

Figure 1 shows the results of the photodynamic treatments at 234 J/cm^2^ against both planktonic cells and biofilms established on silicone rubber (Si), polytetrafluoroethylene (PTFE), stainless steel 316 (SS), and polyethylene terephthalate (PET), as measured by plate counting. Blue light alone (BL+) reduced planktonic cells by 0.6 log CFU/cm^2^, and applying Ca and Cur resulted in an additional reduction of 2.1 and 5.8 log CFU/cm^2^, respectively (*p <* 0.05). BL+ also affected biofilms, but the counts were not decreased by more than 1 log CFU/cm^2^ on SS, PTFE, and Si and 1.7 log CFU/cm^2^ on PET (*p >* 0.05). Ca and Cur significantly enhanced the BL effect (*p <* 0.05). However, we could not achieve the reductions comparable to planktonic cells after BL+ Cur. BL+ Cur was found to be more effective in reducing Lm on PET and PTFE surfaces compared to BL+ Ca. Reduction levels of 1.9 and 2.9 (BL+ Ca) and 3.7 and 3.4 (BL+ Cur) were recorded for PET and PTFE, respectively. We did not find the two statistically different for PTFE. We observed no significant difference in Lm reduction between BL+ Ca and BL+ Cur on Si. The reduction was 2.9 log CFU/cm^2^ for both. BL+ Ca seemed more effective than BL+ Cur in reducing Lm on SS. BL+ Ca and BL+ Cur reduced Lm by 3.7 and 3.0 log CFU/cm^2^, respectively. Of note, Ca alone (BL−) was able to reduce biofilms by c.a. 1.4 log CFU/cm^2^, and in the case of SS, the reduction reached 2 log CFU/cm^2^ (*p <* 0.05). This was not observed with Cur alone (BL−) (Supplementary Figure S1).

### 2.2. Planktonic Cells—FCM Analysis

**Figure 2:**
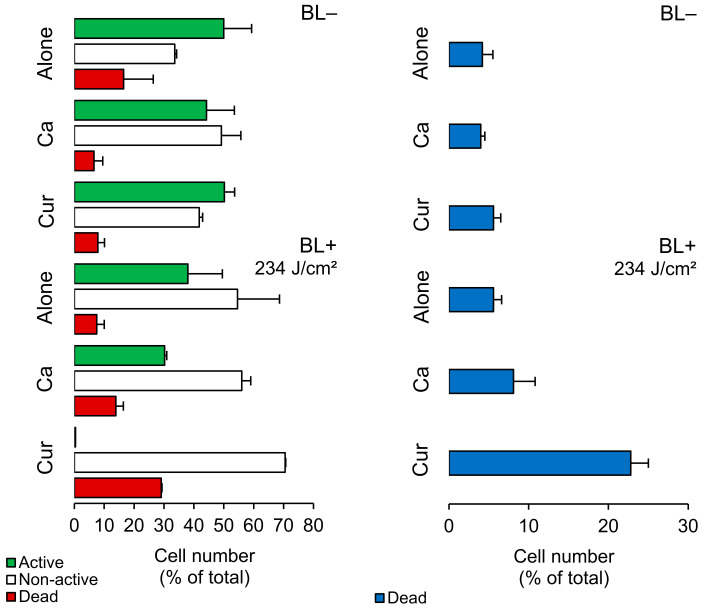
Cell number of *Listeria monocytogenes* ATCC 15313 planktonic cells (% of total; mean ± SD) in *Curcuma* L. (Cur)- and *t*-cinnamaldehyde (Ca)-based (non-)photosensitization experiments (BL−/BL+). Different bar colors indicate different cell subpopulations, as measured by FCM with CFDA/PI (**left**) and TOTO^®^-1 staining (**right**). See Section 4.8 in Materials and Methods for details.

We analyzed planktonic cells using FCM measurements with CFDA/PI staining. Figure 2 shows that BL+ did not harm Lm cells but reduced the number of active cells by 12% and increased non-active cells by 21%. When we applied photosensitizers, Ca and Cur, they caused a 7% and 21% increase in cell damage, respectively. Cur was more effective than Ca in reducing the activity of Lm cells. We further assessed cell damage using TOTO^®^-1 (Figure 2, a membrane-impermeable dye commonly used for this purpose. TOTO^®^-1 staining revealed that BL+ did not cause significant damage, while Ca and Cur increased cell damage by 4% and 17%, respectively. Hence, Ca doubled and Cur quadrupled the damage compared to their BL− counterparts (Supplementary Table S1).

### 2.3. Biofilms—FCM Analysis

**Figure 3:**
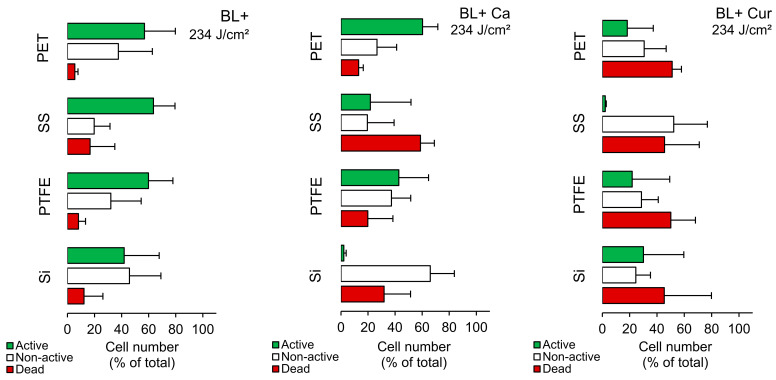
Cell number of *Listeria monocytogenes* ATCC 15313 cells (% of total; mean ± SD) on different surfaces, including silicone rubber (Si), polytetrafluoroethylene (PTFE), stainless steel 316 (SS), and polyethylene terephthalate (PET) in *Curcuma* L. (Cur)- and *t*-cinnamaldehyde (Ca)-based photosensitization experiments (BL+). CF−PI+ cells were labeled as experiencing damage (red bar) and CF+PI− as retaining activity (green bar). CF−PI− cells were labeled as non-active (white bar).

Figure 3 illustrates that when seeded on PET and PTFE, Lm responded similarly to BL+. Both contained fewer dead cells, and the active to non-active cell ratio was close to 60% to 40%. Si emerged with the most non-active cells (46%), while SS had almost 20% dead cells. The same was observed on SS without blue-light exposure (Supplementary Table S2), suggesting SS largely affects Lm fitness. Applying Ca and Cur to PET/PTFE contained, on average, 16% and 50% dead cells, respectively (Figure 3). In the case of Ca, the active cells still constituted the majority. Applying Cur to Si resulted in an almost four-fold increase in cell damage; however, it was inconsistent. With Ca, the percentage of non-active cells was the highest (66%), and active cells were marginal. On SS, Lm responded differently. Ca resulted in 60% dead cells, while Cur produced 45%. At the same time, Cur demonstrated non-active cells at the expense of active cells.

**Figure 4:**
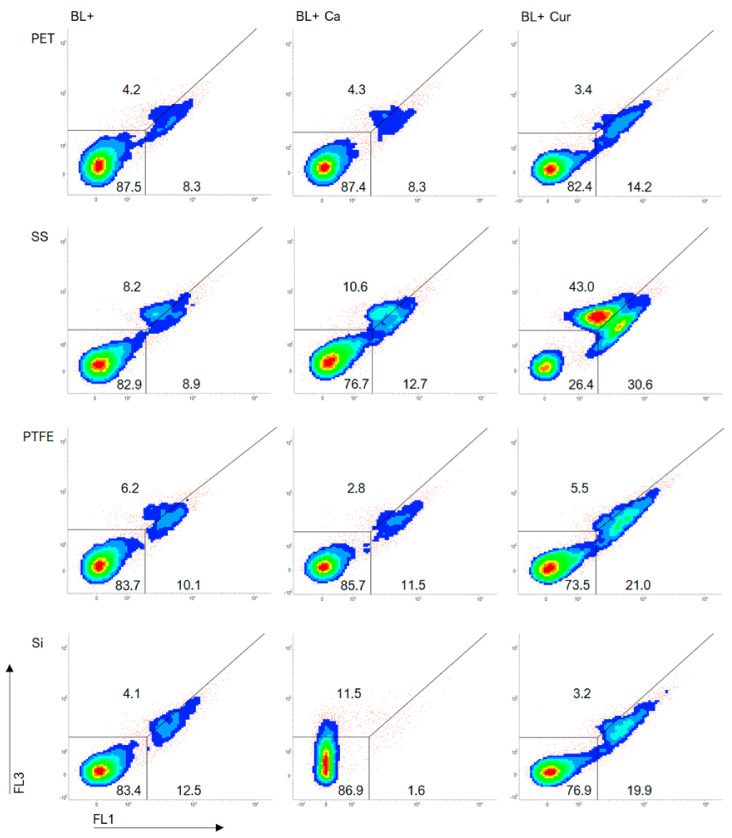
FCM dot plots of *Listeria monocytogenes* ATCC 15313 biofilms established on silicone rubber (Si), polytetrafluoroethylene (PTFE), stainless steel 316 (SS), and polyethylene terephthalate (PET) in *Curcuma* L. (Cur)- and *t*-cinnamaldehyde (Ca)-based photosensitization (BL+) experiments as measured by FCM with TOTO^®^-1 staining.

We included dot plots of TOTO^®^-1 staining to further investigate cell damage to Lm (Figure 4). Supplementary Figure S2 displays the same but without exposure to blue light (BL−). A line across the plots separates dead cells (lower right) from cells exhibiting increased dye permeability (upper right), also considered dead. Photosensitization increased the dead cell population as Cur caused the most cell damage (Figure 4). BL+ Ca and Cur resulted in greater cell damage on SS than on other surfaces. We could also see considerable damage to the cells on the SS in BL− experiments (Supplementary Figure S2). There was no difference in cell damage between BL+ and BL+ Ca on PET and PTFE. BL+ Ca did not impair cells on Si. Although cells may have been impacted, we failed to classify 11.5% of the population (Figure 4).

### 2.4. Adhesion Potential and Hydrophobicity

Both attractive and repulsive forces influence bacterial adhesion to surfaces. Short-range interactions such as polar and apolar interactions are particularly important. Bacterial adhesion becomes more favorable when attractive forces are stronger than repulsive forces [14]. This occurs when the interactions decrease the free energy of adhesion (∆G_adhesion_ < 0). Our results indicate that the free energy of adhesion between the studied Lm strain and Si/SS is positive (∆G_adhesion_ > 0 mJ/m^2^) (Table 1), rendering it thermodynamically unfavorable. The adhesion between the Lm and PET was close to 0 mJ/m^2^. On the other hand, adhesion to PTFE is more likely to occur due to its negative total free energy of adhesion (∆G_adhesion_ < 0 mJ/m^2^).

Considering the values of the water contact angle, Si colonized by the Lm was the most hydrophobic (θ_W_ > 65°). PTFE was close enough to be considered hydrophobic. The last two, SS and PET, were hydrophilic. Variations in θ_W_ occurred more on Si and PTFE because of the random distribution of water drops in areas with varying concentrations of adhered cells. Nonetheless, all surfaces colonized by the Lm were considered hydrophilic because the ∆G^TOT^ values were > 0. The same is suggested by a semi-quantitative measure of hydrophobicity, **γ*_S_*^−^** > 35, and the apolar component **γ*_S_***^LW^ ≤ 45 mJ/m^2^. It is worth noting that the PTFE had the lowest values, while the SS had the highest values.

### 2.5. Principal Component Analysis (PCA)

**Figure 5:**
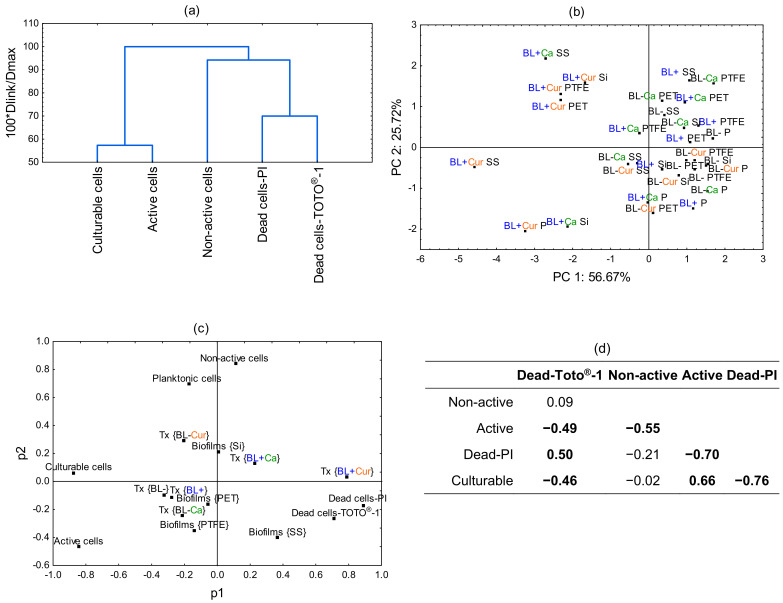
The dissimilarity dendrogram among Lm assayed cells (**a**). The biplot of blue light (BL−/BL+), culture (P−planktonic cells and biofilms on PET, SS, PTFE, and Si), and photosensitization (Cur—*Curcuma* and Ca—*t*-cinnamaldehyde) variables related to PC1 and PC2 (**b**). The loading scatterplot (p1 vs. p2) illustrates clustering among the analyzed variables (**c**). The tested continuous variables include dead cells-PI, dead cells-TOTO^®^-1, active, non-active, and culturable cells. The selected categorical variables were the culture (planktonic cells and biofilms on PET, SS, PTFE, and Si) and the treatment (BL+/BL−; Cur/Ca). The correlation coefficient (r) between differentially assayed cells measures their relationship strength (**d**). The correlation coefficients in bold are significant at a *p* < 0.05. 0—no linear relationship. 0.3—A weak relationship. 0.5—A moderate relationship. 0.7—A strong linear relationship.

PCA was conducted to further resolve how the culture and treatment conditions impact Lm viability. Figure 5a illustrates the dissimilarity measure among the assayed cells. The culturable and active cells are the farthest away from the other cells. Next are the non-active cells, followed by dead cells labeled with PI and TOTO^®^-1. The biplot in Figure 5b shows the separation of Ca and Cur variables with respect to PC1 and PC2. It separates all BL+ Cur treatments, with BL+ Cur SS and BL+ Cur P plotting further away. It also scatters two Ca treatments, BL+ Ca SS and BL+ Ca Si, suggesting cell membrane damage and a loss of activity, respectively. We also examined the loading factor scatterplot and observed significant variable clustering (Figure 5c). Variables placed close to each other influence the PCA model similarly and indicate they are correlated. Dead cells-PI and dead cells-TOTO^®^-1 are variables with a substantial degree of correlation accompanied by BL+ Cur. The next closest variable to them is biofilms SS. There is a strong correlation between planktonic cells and non-active cells, and biofilms Si is the next closest of all biofilms. Other variables were also correlated, e.g., BL+, BL−, and biofilms PET, but they were least influential in determining the PCA model. Finally, we observed a positive correlation between dead cells-TOTO^®^-1 and dead cells-PI (r = 0.50) and active and culturable cells (r = 0.66). On the other hand, we found a negative correlation between the dead cells-TOTO^®^-1 and active/culturable cells (r = −0.49/−0.46), active and non-active cells (r = −0.55), and dead-PI and active/culturable cells (r = −0.70/−0.76) (Figure 5d).

## 3. Discussion

Photodynamic inactivation (PDI) is a highly promising and effective technology that uses light to inactivate harmful microorganisms. This study explored antibacterial and antibiofilm PDI with edible photosensitizers against Lm and focused greatly on evaluating the enzymatic activity and cell damage as part of the antibacterial mechanism. We showed that Ca- and Cur-mediated PDI significantly reduced planktonic cells, reaching 3 and >6 log CFU/cm^2^, respectively. FCM analysis, coupled with CFDA/PI and TOTO^®^-1 staining, indicated that Ca doubled and Cur quadrupled cell damage. In addition, Cur diminished the enzymatic activity of planktonic cells.

Cur has been shown to inhibit Gram-negative and Gram-positive bacteria, with studies primarily focusing on growth and biofilm formation inhibition [15]. This has opened a new avenue for designing environmentally friendly PDI technology. Our results are consistent with the previous reports on curcumin in enhancing PDI against various microorganisms, including those of the *Listeria* genus. Huang et al. [16] achieved almost complete planktonic cell reductions in *Listeria* at 1.0 μM in 5 min. Bonifácio et al. [17] used 3.7 mg/L and 30 min exposure with a total energy of 270 J/cm^2^ to achieve the same effect. This may be due to differences in the solubility of curcumin in various solvents, such as water, acetone, DMSO, and ethanol, as used by different research groups. Furthermore, these variations could be attributed to the differences in the methodology employed, the way the vehicle interacts with the bacterial outer membrane, and the purity level of the curcumin that was utilized in the study.

There is much interest in evaluating the bactericidal efficiency of the curcumin-mediated PDI for food safety purposes. Al-Asmari et al. [9] investigated curcumin photosensitization’s impact on sanitizing fresh date fruit. They found that refrigerated storage had the longest shelf life for fruit treated with curcumin photosensitization (up to 98 days). Liu et al. [18] found that Cur-mediated PDI extended oysters’ shelf life by four days, improving sensory quality without affecting texture and flavor. Tao et al. [19] treated the surface of fresh-cut apple slices with Cur-PDI, which improved reductions in *Escherichia coli*. de Oliveira et al. [10] evaluated the bactericidal activity of Cur at 5 mg/L with PDI against *E. coli* O157:H7 and *L. innocua* in the spinach wash water. After a 5 min exposure, high bacterial reduction was achieved even in high chemical oxygen demand/dirty conditions. de Oliveira et al. [11] conducted a study where they treated the surface of spinach, lettuce, and tomatoes with Cur at a 10 mg/L concentration. The PDI treatment resulted in a 3-log reduction of *E. coli* O157:H7 and *L. innocua* while having no significant effect on the color and texture of the fresh produce. The Cur-PDI technology could also replace fungicides and pesticides as it is effective against various microorganisms, e.g., *Aspergillus flavus* [20]. The use of Cur-mediated PDI has shown promising results in controlling biofilms. Bonifácio et al. [17] tested PDI using Cur against *L. innocua* biofilms and achieved a 4.9-log reduction, which was still less than that of planktonic cells.

In our study, PDI with photosensitizers affected Lm biofilms, but we did not achieve a complete reduction, as the highest was 3.7 log CFU/cm^2^. Cells treated with Ca showed no damage and preserved activity, while Cur-mediated PDI impaired cells on PET and PTFE. In contrast, Ca-mediated PDI diminished the enzymatic activity of cells on Si. SS contained a great number of dead cells, indicating that SS itself impacts Lm viability. In addition, the efficacy of Ca-mediated PDI was enhanced on SS, leading to increased cell damage to Lm. Our recent study found that exposing Lm on SS for 4 h at 668 J/cm^2^ resulted in a less than 1-log CFU/cm^2^ reduction in a dried biofilm model [21]. Gallic acid, used as a photosensitizer, led to an additional 0.5-log CFU/cm^2^ reduction. This demonstrates that Lm is somewhat responsive to photosensitizers.

Reports suggest that PDI of *L. monocytogenes* can occur with different PSs. For instance, Lin et al. [22] obtained almost complete inactivation of planktonic cells with 0.6 mg/L methylene blue and a light dose of 120 J/cm^2^. Given a high inactivation, the use of methylene blue may result in neurotoxicity. Unlike Cur, it is not approved by the US Food and Drug Administration or the European Food Safety Authority as a food additive. Thus, it cannot be considered a suitable PS for food-related applications. The main advantages of using Cur in PDI include its high efficacy as a photosensitizer, as well as its low toxicity. Instead, curcumin can damage the permeability and integrity of bacterial cell membranes, leading to cell death. In addition, curcumin’s lipophilic structure allows it to be directly inserted into the bilayer of liposomes, increasing the permeability of the bilayer [15]. The structure of Cur is likely responsible for increased ROS diffusion across the extracellular matrix and improved efficacy against biofilms of *L. innocua* [17]. In addition, curcumin’s antibiofilm effect may be due to a reduction in the expression of virulence genes, including inlA, hlyA, and *plc*A, which may have the effect of reducing the adhesion and invasive capacity of *L. monocytogenes* [23].

To date, Cur has not been reported as an efficient PS for *L. monocytogenes* on different surfaces under visible light. This opens the perspective of employing this strategy in antimicrobial polymers. For instance, nanoparticles consisting of hyper-crosslinked polymers enable their use in drug delivery [24]. A biodegradable polymer called poly(lactic-co-glycolic acid) (PLGA) has recently been used in combination with curcumin to kill *Staphylococcus bovis* and *Escherichia coli* [25]. This could be further extended to *L. monocytogenes* and employed in the antimicrobial photoactivity of cur-loaded materials, including those recommended for direct food contact or food packaging.

There is no doubt that medicinal plants demonstrate antimicrobial activity, including against foodborne pathogens [26,27,28]. Various effects are expected to occur, including antibiofilm. Phytochemicals affect biofilm formation and reduce listerial biofilms on surfaces commonly used in antibiofilm assays, which are stainless steel or polystyrene. For instance, Upadhyay et al. [29] reported that cinnamaldehyde, at 5 and 10 mM concentrations, can effectively reduce *Listeria* biofilms on SS. Liu et al. [30] found that cinnamaldehyde more effectively inhibited *Listeria* biofilm formation on polystyrene plates than sodium hypochlorite. Although many studies have been on phytochemical efficacy, comparing efficacy on different surfaces has received little attention. One recent study revealed that *trans*-cinnamaldehyde reduced *L. innocua* biofilms by 4.6, 4.0, 3.6, and 2.5 log CFU/cm^2^ from PET, silicone rubber, PTFE, and SS [31]. In contrast to our results, the highest viability of *L. innocua* was on Si and SS, suggesting that each individual *Listeria* interacts with surfaces differently.

We revealed that Lm and Si/SS adhesion is thermodynamically unfavorable. This finding is similar to studies conducted by Bernardes et al. [14] and Teixeira et al. [32], which tested *Bacillus cereus* and *Staphylococcus sciuri*, respectively. Cells were hydrophilic, the material surface was hydrophobic, and the free energy of adhesion was positive. Van Oss [33] explained that bacterial adhesion is facilitated when hydrophobic surfaces come into contact and remove the water between them. However, it is important to note that adhesion can also occur between hydrophobic and hydrophilic surfaces or two hydrophilic surfaces. Since colonization occurred, the Lm could have produced polymers or specific cellular structures called adhesins, contributing to adhesion. Nonetheless, the thermodynamic aspect of adhesion did not favor the interaction between Lm and the two surfaces.

When considering bacterial adhesion and biofilm formation, it is important to consider other/various properties, including the water contact angle of the materials. Our previous study found that Si and PTFE are hydrophobic, while SS and PET are hydrophilic when using a 90° cut-off angle [31]. According to the quantitative criterion (∆G^TOT^), they were all considered hydrophobic. This study classified Si and PTFE as hydrophobic and SS and PET as hydrophilic, but eventually, they were considered hydrophilic because the ∆G^TOT^ values for all were >0 mJ/m^2^. It is because they were colonized by the Lm, and contact angle measurements were related to the hydrophilicity of the cells. θ_W_ and ∆G^TOT^ values suggest SS and PET displayed more contact points for the Lm than Si and PTFE did. Given that SS provided more contact points for the Lm, unfavorable interactions between Lm and SS may have contributed to the increased damage to Lm.

Both antimicrobial blue light and essential oils have broad antimicrobial spectra and a multi-target mode of action. Blue light from 400 to 495 nm kills pathogens by generating ROS that damage cellular components. Essential oils have the ability to affect multiple targets in bacteria, specifically targeting their envelopes [34]. Many are Generally Recognized as Safe (GRAS) and widely used in food preservation, flavoring, fragrance, and cosmetic industries. An innovative strategy for developing more effective interventions against pathogens involves seeking bacteria-specific pro-photosensitizers. Lu et al. [35] recently discovered the potent antimicrobial activity of thymol, which showed strong synergy with blue light against *Staphylococcus aureus* and *Pseudomonas aeruginosa*. The photochemical reaction in bacteria is likely triggered by blue-light excitation of endogenous proporphyrin-like compounds. Likewise, *trans*-cinnamaldehyde exhibits antimicrobial properties and targets multiple bacterial structures. While it showed weaker synergy with blue light than Cur, this study highlights the need for further research and exploration of new phytochemicals that may exhibit higher synergy against *Listeria monocytogenes*.

## 4. Materials and Methods

### 4.1. Bacterium and Culture Conditions

*Listeria monocytogenes* ATCC 15313 originated from the American Type Culture Collection (ATCC™), which is a well-regarded strain in antimicrobial testing experiments [36,37,38]. This strain was cultured overnight at 37 °C on tryptic soy agar (TSA; Merck, Darmstadt, Germany) and an additional 20 h culture at 37 °C in tryptic soy broth (TSB) to obtain stationary-growth-phase cultures.

### 4.2. Planktonic Cells and Biofilms

A stationary-growth-phase culture, with a concentration of 1 × 10^8^ CFU/mL in PBS, was added to a 24-well flat-bottom polystyrene plate (Costar, Corning, NY, USA) in 1000 µL volume along with photosensitizers at final concentrations. The biofilm inocula were prepared at a concentration of 1 × 10^6^ CFU/mL in TSB. The surfaces of the target coupons in the 24-well polystyrene plate were seeded with 1000 µL/well of the inoculum and incubated for one day at 37 °C. The formed biofilms were washed with PBS and then immersed in 1000 µL of photosensitizer solutions.

### 4.3. Material Surfaces

Solid coupon surfaces, each with a diameter of 12.7 cm and a thickness of 3 mm, were used in this study. The coupons were made from different materials, including silicone rubber (Si), polytetrafluoroethylene (PTFE), stainless steel type 316L (SS), and polyethylene terephthalate (PET). These coupons were purchased from BioSurface Technologies, based in Bozeman, MT, USA.

### 4.4. Hydrophobicity and Free Energy of Adhesion

The OCA 15 Plus goniometer (DATAPHYSICS, Filderstadt, Germany) determined the contact angles between Lm surfaces and water, formamide, and α-bromonaphthalene, respectively. In two independent trials, contact angle measurements were taken three times for each. The van Oss approach [39] was used to calculate the hydrophobicity, which is represented by the total free energy of interaction (ΔG_sws_) of the surface when immersed in water (w) (mJ/m^2^). Surface tension elements of the interacting entities can be utilized to conclude ΔG_sws_ as follows (Equation (1)):

(1) $${\Delta G}_{sws}=-2{\left(\sqrt{\gamma_{s}^{LW}}-\sqrt{\gamma_{w}^{LW}}\right)}^{2}+4\left(\sqrt{\gamma_{s}^{+}\gamma_{w}^{-}}+\sqrt{\gamma_{s}^{-}\gamma_{w}^{+}}-\sqrt{\gamma_{s}^{+}\gamma_{s}^{-}}+\sqrt{\gamma_{w}^{+}\gamma_{w}^{-}}\right)$$

where $\gamma^{LW}$ constitutes the Lifshitz–van der Waals component of the surface free energy; $\gamma^{+}$ and $\gamma^{-}$ are the electron acceptor and electron donor parameters of the Lewis acid–base component ($\gamma^{AB}$), with $\gamma^{AB}=2\sqrt{\gamma^{+}\gamma^{-}}$.

Then, three equations of the type below were solved (Equation (2)):

(2) $$\left(1+\cos\theta \right)\;\gamma_{w}^{Tot}=2\left(\sqrt{\gamma_{s}^{LW}\gamma_{w}^{LW}}+\sqrt{\gamma_{s}^{+}\gamma_{w}^{-}}+\sqrt{\gamma_{s}^{-}\gamma_{w}^{+}}\right)$$

where θ is the contact angle and $\gamma^{Tot}=\gamma^{LW}+\gamma^{AB}$. In the end, if the ΔG^TOT^ < 0 mJ/m^2^, the surface is hydrophobic, and if the ΔG^TOT^ > 0 mJ/m^2^, it is hydrophilic.

When studying the interaction between two surfaces, the bacterial cells (b) and food-processing surfaces (s) in aqueous liquid media (l), the total free energy of adhesion (ΔG_adhesion_) can be calculated by solving Equations (3)–(5).

(3) $${\Delta G}_{adhesion}={\Delta G}_{bls}^{LW}+{\Delta G}_{bls}^{AB}$$

(4) $${\Delta G}_{bls}^{LW}=\gamma_{bs}^{LW}-\gamma_{bl}^{LW}-\gamma_{sl}^{LW}$$

(5) $${\Delta G}_{bls}^{AB}=\gamma_{bs}^{AB}-\gamma_{bl}^{AB}-\gamma_{sl}^{AB}$$

where γ_bs_ is the interfacial tension between the bacterial surfaces and the adhesion surface, γ_bl_ is the interfacial tension between the bacterial surfaces and the liquid, and γ_sl_ is the interfacial tension between the adhesion surfaces and the liquid, which can be found in [14]. These equations help determine the energy required to separate the two surfaces, taking into consideration their polarity (${\Delta G}_{bls}^{AB}$) and apolarity (${\Delta G}_{bls}^{LW}$). The ΔG_adhesion_ provides a way to measure the thermodynamic aspects of the adhesion process: if ΔG_adhesion_ < 0 mJ/m^2^, the process is favorable, but if ΔG_adhesion_ > 0 mJ/m^2^, the process is unfavorable.

### 4.5. Phytochemicals

*Curcuma* L. root powder was obtained from Herbapol (Kraków, Poland), and trans-cinnamaldehyde was obtained from Sigma-Aldrich (St. Louise, MO, USA). The photosensitizers were dissolved in dimethyl sulfoxide (DMSO, Sigma-Aldrich). The curcuminoid-rich samples were collected and sonicated in the dark for approx. 5 min. Control experiments were conducted to ascertain the growth inhibitory effects in DMSO (5–10%, *v*/*v*^−1^), and the concentration used in the inactivation experiments was kept below 5%.

### 4.6. Photodynamic Treatments

A 60 min blue-light exposure was conducted using an 18 × 3W commercially available LED array (Epistar Corp.; Hsinchu, Taiwan) with wavelengths of 410, 430, and 460 nm placed 10 cm away from the bottom of the 24-well polystyrene plate containing planktonic cells or biofilms on the target coupons. To measure the light intensity, a power meter with a 400–1050 nm power sensor (ACSE, Kraków, Poland) was used. The light intensity was multiplied by the exposure time in seconds to calculate the emission dose in J/cm^2^. The irradiation was fixed to obtain 234 J/cm^2^ (BL+). We used sub- and inhibitory concentrations of *Curcuma* L. (40 µg/mL) and *trans*-cinnamaldehyde (3 mM), respectively, to test photosensitization efficacy. These concentrations were found in our previous experiment, comprising the minimal inhibitory concentration assay. The photosensitizer solutions were added to the wells of the polystyrene plate, either with planktonic cells or coupons (biofilms), which was then immediately placed under the blue-light source. The bactericidal effects on planktonic and biofilm cells were similarly assessed with BL alone (BL+) and photosensitizers alone. Cells not exposed to light or photosensitizers were used as controls (BL−). Coupons were then transferred onto a new Costar plate. Afterward, they were treated with D/E neutralizing broth (Becton Dickinson, Sparks, MD, USA) for 5 min at room temperature. The coupons were washed using PBS, and then the biofilm-enclosed cells were removed using 1000 µL of PBS through 3 min sonication (Elmasonic SB-120DN; Abchem, Warsaw, Poland). The same neutralization procedure was applied to the planktonic cells after centrifuging at 11,000× *g* for 2 min (MPW 65R, Medical instruments, Warsaw, Poland) and resuspended in PBS. All of them were kept ready for CFU and FCM assays.

### 4.7. CFU Assay

CFU counts of planktonic and biofilm cells were determined via 10-fold serial dilutions on TSA plates and enumerated after 24 h incubation at 37 °C.

### 4.8. FCM Assay

Cells were initially incubated with 50 µM CFDA (5-[and-6-]-carboxyfluorescein diacetate; Sigma-Aldrich), a green cell enzymatic activity marker, delivered in anhydrous dimethyl sulphoxide (Sigma-Aldrich) at 37 °C for 30 min and then 30 µM PI (propidium iodide; Sigma-Aldrich) delivered in double-distilled water, a red nucleic acid probe. The cells were incubated in an ice bath for 10 min. CFDA evaluates intracellular enzymatic activity, as it is hydrolyzed by the cellular esterase into carboxyfluorescein (CF), a membrane-impermeable fluorescent compound. In contrast, PI cannot penetrate cells with intact membranes, indicating cell damage. Consequently, we distinguished cells with diminished activity and compromised membranes (CF−/PI+), referred to as dead, cells that showed intact cells without detectable esterase activity (CF−/PI−), referred to as non-active, and, finally, intact cells preserving enzymatic activity (CF+/PI−), referred to as active. A subset of cells was also incubated with dimeric cyanine nucleic acid stain (TOTO^®^-1) (1 mM solution in DMSO; Invitrogen™ Thermo Fisher, Waltham, MA, USA) at 2 µM. This particular dye has a high affinity for double-stranded DNA and exhibits a significant increase in fluorescence when it binds to nucleic acid. It is a useful indicator for detecting dead cells. TOTO^®^-1 has been shown to produce DNA histograms that offer better results than those obtained with propidium iodide (PI) due to having lower coefficients of variation. The cells were analyzed using a BD FACSLyric™ flow cytometer (Becton Dickinson, San Jose CA, USA), which had two lasers—blue (488 nm, air-cooled, 20 mW solid state) and red (633 nm, 17 mW HeNe). The green fluorescence from the CFDA- and TOTO-1-labeled cells was detected at the FL1 channel (530 ± 30 nm), while the red fluorescence of the PI-labeled cells was detected at the FL3 channel (630 ± 22 nm). To monitor the consistency of the instrument optical alignment, BD™ CS&T Beads (Becton Dickinson) were used, and BD FACSFlow™ solution (Becton Dickinson) was used as the sheath fluid. All bacterial analyses were performed at low rate settings, and a total event count of 50,000 was acquired. The data were analyzed using dot plots, which are bivariate displays where each dot represents one measured event, by the BD FACSSuite V1.3 software (Becton Dickinson).

### 4.9. Statistical Analysis

Mean densities of planktonic cells and biofilms after each photodynamic treatment using *Curcuma* L. and *trans*-cinnamaldehyde were recorded as log CFU/cm^2^. The experiment was repeated three times to ensure accuracy. To analyze the impact of photosensitization among planktonic cells and biofilms, a one-way analysis of variance was performed with Tukey’s test (*p* < 0.05). A Principal Components Analysis (PCA) was conducted to investigate the impact of photosensitization on Lm cells. Hierarchical cluster analysis was performed on cells to determine their dissimilarity extent. The Pearson correlation coefficient (r) was used to evaluate the direction and strength of correlations between assayed cells, with a significance level of *p* < 0.05. All statistical analyses were performed using TIBCO^®^ Statistica™ ver. 13.3 (TIBCO Software Inc., Tulsa, OK, USA).

## 5. Conclusions

Curcumin-rich extracts have demonstrated excellent photosensitizing properties against both *Listeria monocytogenes* planktonic cells and biofilms. The effectiveness of Ca was comparatively lower than that of Cur. However, Ca has potent antibiofilm effects that vary depending on the surface on which Lm resides. This study may help in identifying more effective plant-based compounds to combat *L. monocytogenes* in an environmentally sustainable way.

## Figures and Tables

**Table 1:** Free energy of adhesion (ΔG_adhesion_) between *L. monocytogenes* ATCC 15313 (b) and surfaces (s): silicone rubber (Si), polytetrafluoroethylene (PTFE), stainless steel 316 (SS), and polyethylene terephthalate (PET) in aqueous liquid media (l) and their apolar (ΔG_bls_^LW^) and polar (ΔG_bls_^AB^) components; supplemented with contact angles, surface tension components, and total free energy of interaction (ΔG_sws_^TOT^) of the Lm surfaces.

	Free Energy of Adhesion (mJ/m^2^)			Contact Angles (°) *			Surface Tension Components (mJ/m^2^)				ΔG^TOT^ (mJ/m^2^)
Surface	ΔG_bls_^LW^	ΔG_bls_^AB^	ΔG_adhesion_	θ_W_	θ_F_	θ_B_	γ*_S_*^LW^	γ*_S_*^+^	γ*_S_*^−^	γ*_S_*^AB^	
PET	3.4	3.4	0.0	57.0 ± 18.6	71.2 ± 4.3	65.2 ± 14.3	22.3	0	49.9	0	40.7
SS	−3.2	6.2	2.9	53.2 ± 17.9	67.2 ± 3.1	54.5 ± 14.5	27.7	0	52.4	0	43.5
PTFE	0.0	−4.4	−4.4	63.0 ± 36.1	73.5 ± 10.2	59.8 ± 6.0	25.1	0	41.7	0	28.2
Si	2.4	1.3	3.7	70.8 ± 38.9	88.1 ± 10.6	59.6 ± 20.7	25.2	0	46.1	0	34.9

θ_W_, the contact angle of water; θ_F_, the contact angle of formamide; θ_B_, the contact angle of α-bromonaphthalene. γ_S_^LW^, apolar surface energy component; γ_S_+, electron acceptor surface energy component; γ_S_^−^, electron donator surface energy component; γ_S_^AB^, polar surface energy component; ΔG_adhesion_ < 0 mJ/m^2^ is thermodynamically favorable; ΔG_adhesion_ > 0 mJ/m^2^ is thermodynamically unfavorable. ΔG^TOT^, hydrophobicity degree. * Mean values ± standard deviation.

## Data Availability

The raw data supporting the conclusions of this article will be made available by the authors on request.

## Supplementary Materials

The following supporting information can be downloaded at: https://www.mdpi.com/article/10.3390/molecules29030685/s1, Figure S1: Cell numbers of *Listeria monocytogenes* ATCC 15313 planktonic cells and biofilms established on silicone rubber (Si), polytetrafluoroethylene (PTFE), stainless steel 316 (SS), and polyethylene terephthalate (PET) in *Curcuma* L. (Cur)- and *t*-cinnamaldehyde (Ca)-based non-photosensitization (BL−) experiments; Table S1: Percent of *Listeria monocytogenes* ATCC 15313 planktonic cells (% of total) in *Curcuma* L. (Cur)- and *t*-cinnamaldehyde (Ca)-based (non-)photosensitization experiments as measured by FCM with CFDA/PI and TOTO^®^-1 staining; Table S2: Percent of *Listeria monocytogenes* ATCC 15313 biofilms established on silicone rubber (Si), polytetrafluoroethylene (PTFE), stainless steel 316 (SS), and polyethylene terephthalate (PET) in *Curcuma* L. (Cur)- and *t*-cinnamaldehyde (Ca)-based non-photosensitization (BL−) experiments as measured by FCM with CFDA/PI; Figure S2: FCM dot plots of *Listeria monocytogenes* ATCC 15313 biofilms established on silicone rubber (Si), polytetrafluoroethylene (PTFE), stainless steel 316 (SS), and polyethylene terephthalate (PET) in Curcuma L. (Cur)- and t-cinnamaldehyde (Ca)-based non-photosensitization (BL−) experiments as measured by FCM with TOTO^®^-1 staining.
