# Supplementary material for: Antibacterial and Antibiofilm Effects of Photodynamic Treatment with Curcuma L. and Trans-Cinnamaldehyde against Listeria monocytogenes
Source: Molecules. 2024 Feb 1;29(3):685. doi: 10.3390/molecules29030685 (PMC10856099; doi:10.3390/molecules29030685)
Supplement: Supplementary file 1 [file molecules-29-00685-s001.zip › molecules-2762809-supplementary.pdf]

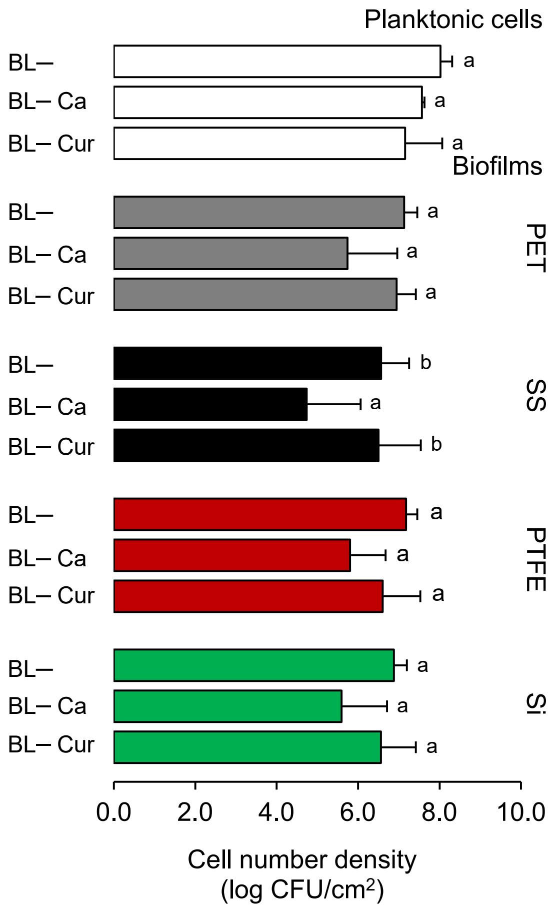

Supplementary Figure S1. Cell numbers of *Listeria monocytogenes* ATCC 15313 planktonic cells and biofilms established on silicone rubber (Si), polytetrafluoroethylene (PTFE), stainless steel 316 (SS), and polyethylene terephthalate (PET) in *Curcuma* L. (Cur)- and *t*-cinnamaldehyde (Ca)-based non-photosensitization (BL–) experiments.

Supplementary Table S1. Percent of *Listeria monocytogenes* ATCC 15313 planktonic cells (% of total) in *Curcuma* L. (Cur)- and *t*-cinnamaldehyde (Ca)-based (non-)photosensitization experiments as measured by FCM with CFDA/PI and TOTO®-1 staining.

		CFDA/PI						TOTO®-1	
		BL–			BL+			BL–	BL+
	**Cells**	**Dead**	**Non-active**	**Active**	**Dead**	**Non-active**	**Active**	**Dead**	**Dead**
**Tx**	**Alone**	16.5±9.9	33.6±0.6	50.0±9.3	7.5±2.5	54.6±14.0	38.0±11.5	4.2±1.3	5.6±1.0
	**Ca**	6.6±2.9	49.2±6.5	44.2±9.3	13.9±2.5	56.0±3.0	30.2±0.7	4.0±0.5	8.1±2.7
	**Cur**	7.9±2.2	41.8±1.1	50.2±3.4	29.1±0.2	70.5±0.2	0.4±0.0	5.6±0.9	22.8±2.2

Supplementary Table S2. Percent of *Listeria monocytogenes* ATCC 15313 biofilms established on silicone rubber (Si), polytetrafluoroethylene (PTFE), stainless steel 316 (SS), and polyethylene terephthalate (PET) in *Curcuma* L. (Cur)- and *t*-cinnamaldehyde (Ca)-based non-photosensitization (BL–) experiments as measured by FCM with CFDA/PI.

	**BL–**			**BL–** Ca			**BL–** Cur		
	**Dead**	**Non-active**	**Active**	**Dead**	**Non-active**	**Active**	**Dead**	**Non-active**	**Active**
**PET**	9.8	41.5	48.7	27.5	26.1	46.5	12.1	57.4	30.5
**SS**	20.5	29.6	49.9	12.3	46.2	41.6	22.5	44.1	33.4
**PTFE**	4.9	43.3	50.1	9.2	20.1	70.8	9.0	41.5	49.4
**Si**	4.7	42.6	52.7	10.0	33.7	56.2	10.4	46.4	43.2

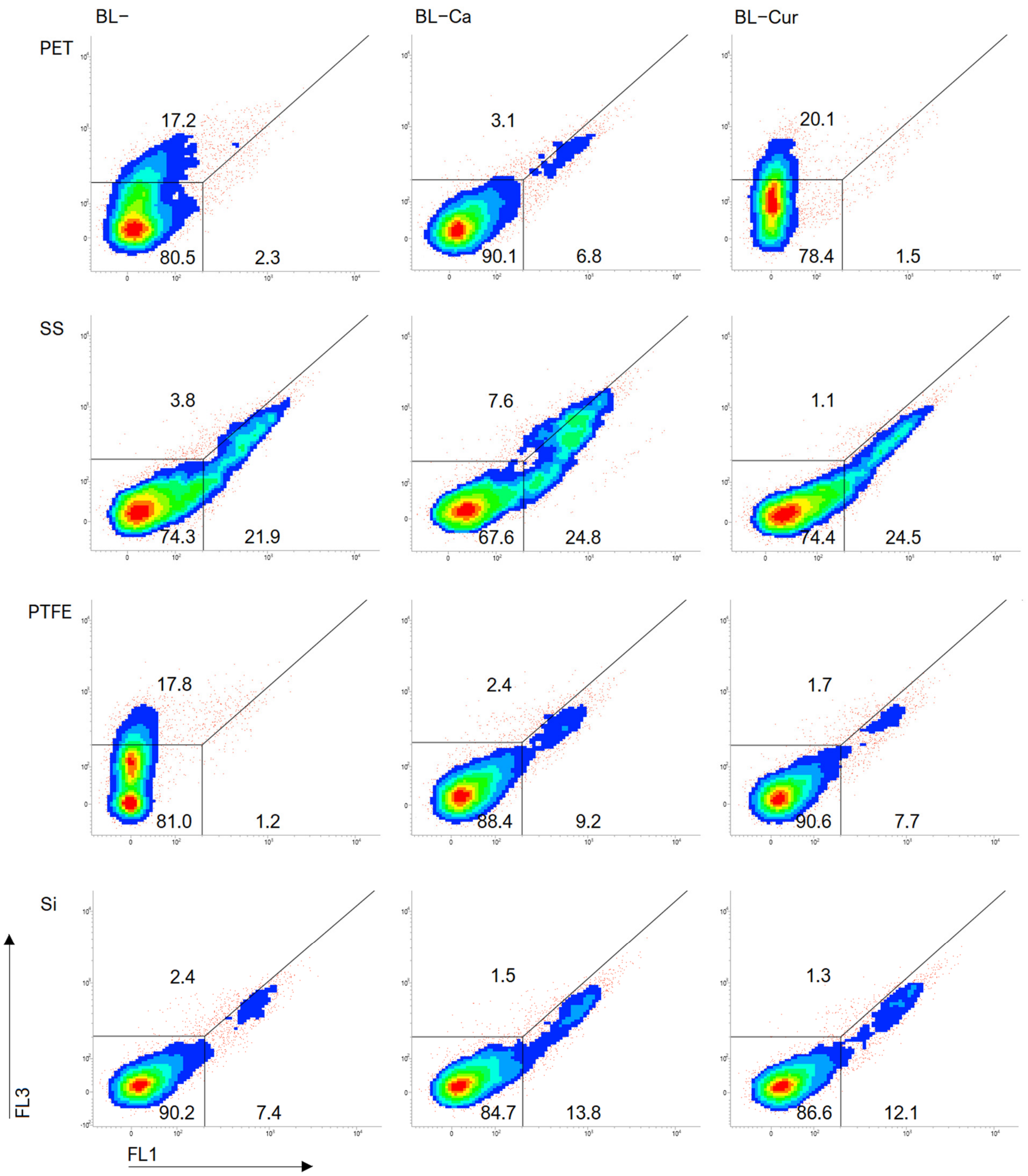

Supplementary Figure S2. FCM dot plots of *Listeria monocytogenes* ATCC 15313 biofilms established on silicone rubber (Si), polytetrafluoroethylene (PTFE), stainless steel 316 (SS), and polyethylene terephthalate (PET) in *Curcuma* L. (Cur)- and *t*-cinnamaldehyde (Ca)-based non-photosensitization (BL—) experiments as measured by FCM with TOTO®-1 staining.
